# Risk analysis and assessment based on Sigma metrics and intended use

**DOI:** 10.11613/BM.2018.020707

**Published:** 2018-06-15

**Authors:** Yong Xia, Hao Xue, Cunliang Yan, Bowen Li, ShuQiong Zhang, Mingyang Li, Ling Ji

**Affiliations:** 1 Department of Clinical Medical Laboratory, Peking University Shenzhen Hospital, Futian District, Shenzhen, China; 2 Student, Guangdong Medical University, Dongguan, China

**Keywords:** risk analysis, risk assessment, Six Sigma, Sigma metrics, failure mode and effects analysis

## Abstract

**Introduction:**

In order to ensure the quality in clinical laboratories and meet the low risk requirements of patients and clinicians, a risk analysis and assessment model based on Sigma metrics and intended use was constructed, based on which differential sigma performance (*σ*) expectations of 42 analytes were developed.

**Materials and methods:**

Failure mode and effects analysis was applied to produce an analytic risk rating based on three factors, each test of which was graded as follows: 1) Sigma metrics; 2) the severity of harm; 3) intended use. By multiplying the score of Sigma metrics by the score of severity of harm by the score of intended use, each was assigned a typical risk priority number (RPN), with RPN ≤ 25 rated as low risk. Low risk was defined as acceptable standards; the sigma performance expectations were calculated.

**Results:**

Among the 42 analytes, tests with *σ* ≥ 6, 5 ≤ *σ* < 6, 4 ≤ *σ* < 5, 3 ≤ *σ* < 4, *σ* < 3 were 21, 5, 5, 6, and 5, respectively; there were 7 high-risk tests, 8 of them medium risk tests. According to the risk assessment conclusion, 13 tests had sigma performance expectations ≥ 6; 15 test items had sigma performance expectations ≥ 5, while 3 test items had sigma performance expectations ≥ 4; 11 test items had sigma performance expectations ≥ 3.

**Conclusions:**

Constructing the risk analysis and assessment model based on Sigma metrics and intended use will help clinical laboratories to identify the high-risk tests more objectively and comprehensively. Such model can also be used to establish the sigma performance expectations and meet the low risk requirements of patients and clinicians.

## Introduction

Laboratories have a major impact on patient safety, as 80 – 90% of all diagnoses are made on the basis of laboratory tests (*1*). Laboratory errors have a frequency of 0.012 – 0.6% for all test results (*2*). A series of regulatory requirements and practice guidelines have been introduced to guide the establishment and continuous improvement of the quality management system to reduce the risk of the total testing process (*3*-*5*).

Failure mode and effects analysis (FMEA), one of the most proactive methods of risk management, has been accepted as the method of choice in the identification of potential points of failure within a process, their effects being determined and action identified for mitigating failures (*6*). The first step in FMEA is to identify all potential possible failure modes of the product or system. After that, critical analysis is performed on these failure modes and the risk priority number (RPN) is calculated by the multiplication of the occurrence (O), severity (S) and detection (D). Finally, the failure modes can be ranked and then proper actions will be preferentially taken on the high-risk failure modes (*7*). The application of the FMEA tool is consistent with the risk-based thinking required by ISO 9001 in the critical decisions, and plays an important role in ensuring the reliability of the product system. In contrast, Shebl *et al.* conducted numerous interviews with hospital staff in the United Kingdom and concluded: “FMEA in health care is associated with a lack of standardization in how the scoring scales are used and how failures are prioritized.” (*8*). Different technicians and different scoring methods yielded dissimilar results; it is a tool for which there is a lack of evidence (*9*). The Clinical and Laboratory Standards Institute (CLSI) EP23A guideline: Laboratory Quality Control Based on Risk Management provides an introduction to risk management techniques and guidance on developing a risk-based quality control plan (QCP) (*3*). The 2-factor model that includes only the probability of occurrence of harm and the severity of harm, does not consider the detection capability, is not conducive to the development of a robust laboratory QCP (*10*). Six Sigma is a technique that allows objective assessment of process performance. The resulting RPN on a sigma-scale is more objective, because it is less reliant on subjective rankings and more reliant on observed performance (*11*). Six sigma quality control (QC) design tools can enhance FMEA, the risk assessment process and design of QC plans (*11*).

In this study, a risk analysis and assessment model based on Sigma metrics and intended use was constructed to ensure the quality in clinical laboratories and meet the low risk requirements of patients and clinicians.

## Materials and methods

### Materials

This study was performed in the Clinical Chemistry Laboratory of the Peking University Shenzhen Hospital, Shenzhen, China, in 2017. The laboratory is accredited according to the International Organization for Standardization (ISO) 15189 2012 by China National Accreditation Service for Conformity Assessment in 2015. Thirty-six serum analytes were evaluated on the Beckman AU5800 chemistry analyser (Beckman Coulter, Tokyo, Japan), which included 27 original manufacturer reagents and 9 “non-kit” reagents. The 27 original manufacturer reagents were: alanine aminotransferase (ALT), aspartate aminotransferase (AST), alkaline phosphatase (ALP), gamma-glutamyltransferase (GGT), total protein (TP), albumin (Alb), total bilirubin (BT), direct bilirubin (BD), urea, creatinine (CREA), uric acid (UA), glucose (Glc), creatinine kinase (CK), lactate dehydrogenase (LD), amylase (AMY), triglycerides (TG), total cholesterol (CHOL), high density lipoprotein cholesterol (HDL), low density lipoprotein cholesterol (LDL), potassium (K), sodium (Na), chloride (Cl), calcium (Ca), magnesium (Mg), inorganic phosphate (Phos), iron (Fe), and transferrin (TRSF). There were 9 “non-kit” reagents, of which IgG, IgM, IgA, pre-albumin (PA), cystatin C (Cys-C) were from DiaSys (Holzheim, Germany); ß2-microglobulin (BMG), C3, C4 were from Leadman (Beijing, China) and C-reactive protein (CRP) was from Sekisui (Tokyo, Japan). Johnson Vitros5600 analyser (Ortho Clinical Diagnostics, Raritan, USA) and original manufacturer reagents were used to measure cardiac troponin I (cTn-I), Roche e601 analyser (Roche Diagnostics, Mannheim, Germany) and original manufacturer reagents for cardiac troponin T (cTn-T), Siemens RP500 blood gas analyser (Siemens Healthcare Diagnostics, Suffolk, United Kingdom) and original manufacturer reagents for pH, pCO_2_, pO_2_ and SEBIA CAPILLARY 2 capillary electrophoresis analyser and original manufacturer reagents (Sebia, Evrycedex, France) for HbA1c. Some commercial control samples of human origin were used for evaluation. Unassayed Specialty Chemistry & Protein Control level 1 and 2 (Qualab Biotech, Shanghai, China) were used for Cys-C. Liquid Unassayed Special Protein Control level 2 and 3 (Qualab Biotech, Shanghai, China) were used for PA, BMG, CRP. Liquichek Cardiac Markers Plus Control LT level 1 and 2 (Bio-Rad Laboratories, Irving, USA) were used for cTn-I, cTn-T. Rapid QC Complete level 1, 2 and 3 (Siemens Healthcare Diagnostics Inc, Terry town, USA) were used for pH, pCO2, pO2; HbA1c capillary controls (expected values-HbA1c percentages) (Sebia, Lisses, France) were used for HbA1c. For all other tests Liquid Assayed Multiqual level 1, 2 and 3 (Bio-Rad Laboratories, Irving, CA, USA) were used.

### Methods

#### The critical decision level-making Sigma metrics

The critical decision levels were determined from a sigma verification program (SVP) (Westgard QC, Madison, USA) or established upon consultation with clinicians. Imprecision was calculated from cumulative results of the internal quality control (IQC) excluded out-of-control in different time periods of 2016 (Table 1). Cumulative coefficient of variation (CV) or standard deviation (SD) was chosen from QC with the concentration closest to the level of critical decision marked in the CV% column of Table 1. Bias calculations were from 2016 external quality assurance (EQA) programs in laboratory medicine by National Centre of Clinical Laboratories (NCCL) of China. Passing-Bablok regression was performed using Microsoft Excel 2007 to determine the bias between the test result and the instrument group mean for comparison from the ten or fifteen EQA program results for each test (*12*). The equation generated y = ax + b (R^2^ > 0.95) was then applied to determine bias at the critical decision level. Total allowable error (TEa) referred to the SVP and EQA criteria from NCCL of China. The Sigma metrics were calculated as follows: Sigma metrics = (TEa - bias) / SD or Sigma metrics = (TEa% - bias%) / CV%.

**Table 1:** Sigma metrics at the critical decision levels and risk analysis and assessment

**Test/unit**	**CDL**	**Tea (source)**	**Regression equation** **[y = ax (95% CI) +** **b (95% CI)]**	**Bias** **(%)**	**CV** **(%)**	**N**	**Sigma**	**Score**	**Severity**	**Score**	**Intended use**	**Score**	**RPN**	**RAA**
**ALT (U/L)**	95^SVP^	20% (CLIA)	y = 0.98 (0.97 to 0.99) x - 0.86 (- 2.36 to 0.64)	- 3.0	2.69^2^	282*	6.3	1	Serious	3	S/M	4	12	low
**AST (U/L)**	40^SVP^	20% (CLIA)	y = 0.95 (0.93 to 0.96) x - 1.23 (- 3.64 to 1.18)	- 8.2	1.71^2^	283*	6.9	1	Serious	3	S/M	4	12	low
**GGT (U/L)**	85^SVP^	22.1% (RD)	y = 0.98 (0.97 to 1.00) x + 0.39 (- 2.41 to 3.19)	- 1.1	1.52^2^	279*	13.8	1	Minor	2	S/M	4	8	low
**ALP (U/L)**	150^SVP^	30% (CLIA)	y = 0.87 (0.82 to 0.93) x + 3.30 (- 7.22 to 13.81)	- 10.4	2.36^2^	280*	8.3	1	Minor	2	S/M	4	8	low
**TP (g/L)**	57^SVP^	10% (CLIA)	y = 1.00 (0.98 to 1.01) x + 0.37 (- 0.61 to 0.98)	0.3	1.43^2^	286*	6.8	1	Minor	2	M	3	6	low
**ALB (g/L)**	25^SVP^	10% (CLIA)	y = 0.94 (0.93 to 0.95) x + 3.09 (2.66 to 3.53)	6.7	1.49^2^	278*	2.2	5	Minor	2	M	3	30	middle
**BT (µmol/L)**	51.3^SVP^	20% (CLIA)	y = 1.04 (1.03 to 1.05) x – 2.88 (- 3.64 to – 2.12)	- 1.4	1.36^2^	286*	13.7	1	Critical	4	S/M	4	16	low
**BD (µmol/L)**	5.13^SVP^	44.5% (RD)	y = 1.00 (0.93 to 1.07) x – 0.69 (- 2.77 to 1.38)	- 13.4	2.87^1^	285*	10.8	1	Serious	3	S/M	4	12	low
**Urea (mmol/L)**	14.28^SVP^	9% (CLIA)	y = 1.00 (0.98 to 1.02) x + 0.002 (- 0.26 to 0.27)	0.1	1.74^2^	287*	5.1	2	Serious	3	S/M	4	24	low
**CREA (µmol/L)**	176.8^SVP^	15% (CLIA)	y = 1.00 (0.99 to 1.01) x – 0.53 (- 3.87 to 2.85)	0.2	1.69^2^	284*	8.8	1	Critical	4	D	5	20	low
**UA (µmol/L)**	196.3^SVP^	17% (CLIA)	y = 0.99 (0.95 to 0.99) x – 6.21 (- 13.80 to 1.38)	- 5.4	1.59^2^	281*	7.3	1	Serious	3	D	5	15	low
**Glc (mmol/L)**	6.66^SVP^	10% (CLIA)	y = 0.99 (0.97 to 1.00) x – 0.01 (- 0.18 to 0.15)	- 1.5	1.94^2^	285*	4.4	3	Critical	4	D/S/M	5	60	high
**CK (U/L)**	275^SVP^	30% (CLIA)	y = 1.01 (0.98 to 1.05) x – 4.34 (- 18.86 to 10.18)	- 0.2	2.57^2^	281*	11.6	1	Serious	3	S/M	4	12	low
**Test/unit**	**CDL**	**TEa (source)**	**Regression equation** **[y = ax (95% CI) +** **b (95% CI)]**	**Bias** **(%)**	**CV** **(%)**	**N**	**Sigma**	**Score**	**Severity**	**Score**	**Intended use**	**Score**	**RPN**	**RAA**
**LDH (U/L)**	170^SVP^	20% (CAP PT)	y = 0.99 (0.96 to 1.02) x + 1.06 (- 4.65 to 6.77)	- 0.2	3.15^2^	278*	6.3	1	Minor	2	S/M	4	8	low
**AMY (U/L)**	145^SVP^	30% (CAP PT)	y = 0.88 (0.78 to 0.98) x + 8.65 (- 17.79 to 35.09)	- 6.1	1.86^2^	280*	12.8	1	Critical	4	D/M	5	20	low
**TG (mmol/L)**	1.47^SVP^	25% (CLIA)	y = 0.93 (0.90 to 0.96) x + 0.04 (- 0.01 to 0.08)	- 4.8	1.41^2^	279*	14.3	1	Minor	2	M	3	6	low
**CHOL (mmol/L)**	4.66^SVP^	10% (CLIA)	y = 1.00 (0.98 to 1.01) x – 0.01 (- 0.80 to 0.05)	- 0.9	1.85^2^	288*	4.9	3	Minor	2	M	3	18	low
**HDL-C (mmol/L)**	1.30 ^SVP^	30% (CLIA)	y = 0.95 (0.97 to 0.92) x + 0.05 (0.01 to 0.10)	- 1.3	3.54^2^	279*	8.1	1	Negligible	1	M	3	3	low
**LDL-C (mmol/L)**	2.59^SVP^	20% (CLIA)	y = 1.10 (0.96 to 1.23) x – 0.34 (- 0.82 to 0.15)	- 3.3	2.13^2^	280*	7.8	1	Minor	2	M	3	6	low
**K (mmol/L)**	2.5^SVP^	0.5 (CLIA)	y = 0.98 (0.96 to 1.00) x + 0.08 (- 0.01 to 0.17)	0.04	0.03^1^ (SD)	288*	15.4	1	Catastrophic	5	D/M	5	25	low
**Na (mmol/L)**	115^SVP^	4 (CLIA)	y = 1.00 (0.95 to 1.06) x – 0.54 (- 8.01 to 6.94)	- 0.2	1.21^1^ (SD)	290*	3.1	4	Critical	4	D/M	5	80	high
**Cl (mmol/L)**	100^SVP^	5% (CLIA)	y = 1.02 (1.00 to 1.05) x – 2.69 (- 5.02 to – 0.37)	- 0.3	1.18^2^	288*	4.0	3	Serious	3	M	3	27	middle
**Ca (mmol/L)**	3.25^SVP^	0.25 (CLIA)	y = 0.95 (0.92 to 0.97) x + 0.11 (0.04 to 0.18)	- 0.06	0.04^3^ (SD)	289*	4.7	3	Critical	4	D/M	5	60	high
**Mg (mmol/L)**	1.03^SVP^	25% (CAP PT)	y = 0.94 (0.92 to 0.96) x + 0.05 (0.03 to 0.07)	- 1.6	2.18^2^	287*	10.7	1	Serious	3	M	3	9	low
**Phos (mmol/L)**	1.29^SVP^	10.7% (CAP PT)	y = 0.98 (0.96 to 1.00) x + 0.04 (- 0.00 to 0.07)	1.06	1.51^2^	284*	6.4	1	Serious	2	M	3	6	low
**Fe (µmol/L)**	13.43^SVP^	20% (CLIA)	y = 0.98 (0.96 to 1.00) x + 0.82 (0.17 to 1.47)	4.1	1.32^2^	276*	12.0	1	Minor	2	D	5	10	low
**TRSF (g/L)**	3.6^SVP^	20% (CAP PT)	y = 1.05 (1.00 to 1.10) x + 0.01 (- 0.11 to 0.12)	5.1	1.72^3^	193*	8.7	1	Minor	2	D	5	10	low
**IgG (g/L)**	7^SVP^	25% (CLIA)	y = 1.00 (0.93 to 1.08) x + 0.03 (- 1.02 to 1.08)	0.9	4.65^3^	290*	5.2	2	Minor	2	M	3	12	low
**IgM (g/L)**	0.6^SVP^	16.8% (RD)	y = 1.00 (0.96 to 1.04) x – 0.02 (- 0.07 to 0.02)	- 3.8	2.53^1^	285*	5.1	2	Minor	2	M	3	12	low
**IgA (g/L)**	0.6^SVP^	13.5% (RD)	y = 1.01 (0.99 to 1.02) x – 0.03 (- 0.09 to 0.02)	- 4.4	2.46^1^	286*	3.7	4	Minor	2	M	3	24	low
**C3 (g/L)**	1.35^SVP^	8.4% (RD)	y = 1.07 (1.02 to 1.12) x – 0.06 (- 0.14 to 0.01)	2.5	3.19^3^	288*	1.9	5	Serious	3	M	3	45	middle
**C4 (g/L)**	0.75^SVP^	16% (RD)	y = 1.05 (0.97 to 1.13) x – 0.00 (- 0.02 to 0.02)	4.2	3.41^3^	286*	3.5	4	Serious	3	M	3	36	middle
**PA (mg/L)**	150^SVP^	14.5% (RD)	y = 0.92 (0.83 to 1.01) x + 18.64 (0.47 to 36.8)	4.5	2.34^2^	198^†^	4.3	3	Negligible	1	M	3	9	low
**BMG (mg/L)**	2.7^SVP^	9% (RD)	y = 1.09 (1.00 to 1.18) x – 0.05 (- 0.38 to 0.27)	6.9	3.4^2^	190^†^	0.6	5	Serious	3	S/M	4	60	high
**Cys-C (mg/L)**	10^SVP^	7.6% (RD)	y = 1.02 (0.97 to 1.07) x – 0.02 (- 0.28 to 0.23)	1.8	1.8^1^	191^‡^	3.2	4	Serious	3	S/M	4	48	middle
**CRP (mg/L)**	30^SVP^	25% (NCCL)	y = 0.92 (0.89 to 0.95) x + 0.40 (- 0.97 to 1.78)	- 6.9	2.18^2^	195^†^	8.3	1	Negligible	1	M	3	3	low
**cTn-I (ng/mL)**	0.4^cc^	30% (NCCL)	y = 1.02 (0.98 to 1.05) x – 0.04 (- 1.08 to 1.00)	- 8.2	4.34^1^	195^§^	5.0	2	Catastrophic	5	D	5	50	middle
**cTn-T (ng/mL)**	0.1^cc^	30% (NCCL)	y = 0.96 (0.93 to 0.98) x – 0.00 (- 0.04 to 0.04)	- 5.5	6.52^1^	354^║^	3.8	4	Catastrophic	5	D	5	100	high
**Test/unit**	**CDL**	**TEa (source)**	**Regression equation** **[y = ax (95% CI) +** **b (95% CI)]**	**Bias** **(%)**	**CV** **(%)**	**N**	**Sigma**	**Score**	**Severity**	**Score**	**Intended use**	**Score**	**RPN**	**RAA**
**pH**	7.1^cc^	0.04 (NCCL)	y = 1.05 (1.03 to 1.07) x – 0.39 (- 0.53 to - 0.24)	- 0.02	0.004^1^ (SD)	166^¶^	5.8	2	Critical	4	D	5	40	middle
**pCO2 (mmHg)**	75^cc^	8% (NCCL)	y = 1.01 (0.96 to 1.05) x – 0.58 (- 3.03 to 1.86)	- 0.2	2.29^1^	165^¶^	3.4	4	Critical	4	D	5	80	high
**pO2 (mmHg)**	80^cc^	8% (NCCL)	y = 0.91 (0.86 to 0.97) x + 12.27 (6.91 to 17.64)	6.7	5.58^2^	167^¶^	0.2	5	Critical	4	D	5	100	high
**HbA1c (%)**	6.5^NGSP^	7% (NCCL)	y = 1.04 (1.00 to 1.08) x – 0.37 (- 0.41 to - 0.33)	- 2.1	1.73^1^	106**	2.8	5	Serious	3	M	3	45	middle
CDL - critical decision levels. TEa - total allowable error. CV – coefficient of variation. RAA - risk analysis and assessment. RPN - risk priority number . SVP - Sigma verification program. CLIA - Clinical Laboratory Improvement Amendments. S – screening. M - patient management decisions. RD - Ricos desirable. CC - clinical consultation. NGSP - National Glycohaemoglobin Standardization Program. CAP - College of American Pathologists. PT - proficiency testing. NCCL - National Centre of Clinical Laboratories of China External Quality Assurance. D – diagnosis. SD – standard deviation. ALT - alanine aminotransferase. AST - aspartate aminotransferase. ALP - alkaline phosphatase. GGT - gamma-glutamyltransferase. TP - total protein. ALB – albumin. BT – bilirubin, total. BD – bilirubin, direct. CREA – creatinine. UA - uric acid. Glc – glucose. CK - creatinine kinase. LD - lactate dehydrogenase. AMY – amylase. TG – triglycerides. CHOL - cholesterol. HDL – high density lipoprotein. LDL – low density lipoprotein. K – potassium. Na – sodium. Cl – chloride. Ca – calcium. Mg – magnesium. Phos – inorganic phosphate. Fe – iron. TRSF - transferrin. IgG – immunoglobulin G. IgM – immunoglobulin M. IgA – immunoglobulin A. PA - pre-albumin. Cys-C - cystatin C. BMG - ß2-microglobulin. C3 – complement C3. C4 – complement C4. CRP - C-reactive protein. cTn-I – cardiac troponin I. cTn-T – cardiac troponin T. pH – acidity. pCO2 - partial pressure of carbon dioxide. pO2 - partial pressure of oxygen. HbA1c – glycated haemoglobin. CV% column: the superscript ^1,2,3^ indicate the CV% determined from the IQC level 1, level 2 or level 3, respectively. N column: *indicate the imprecision calculated from 10 months (March - December 2016), ^†^indicate the imprecision calculated from 9 months (April - December 2016), ^‡^indicate the imprecision calculated from 9 months (January - September 2016), ^§^indicate the imprecision calculated from 7 months (June - December 2016), ^║^indicate the imprecision calculated from 11 months (February - December 2016), ^¶^indicate the imprecision calculated from 6 months (April - September 2016), **indicate the imprecision calculated from 3 months (April - June 2016).														

#### Risk assessment based on Sigma metrics and intended use

The severity of harm due to exceeding TEa was investigated *via* a questionnaire survey through the Internet (www.wenjuan.com). Construction of questionnaire survey referred the manuscript of “Guidelines for constructing a survey” (*13*). In the questionnaire, total 42 questions were designed for 42 analytes, and the severity of harm was defined at the level of medical critical decision. For example, “ALT (test name) results deviate from the true results of more than 20% (TEa) at the level of 95U/L (critical decision level), how do you think the impact on clinical diagnosis and treatment? Neglected, minor, serious, critical, catastrophic”. The questionnaire was published in three WeChat work groups in where are 44 doctors in different clinical departments of Peking University Shenzhen hospital, 22 laboratory technicians in Peking University Shenzhen hospital and 23 clinical biochemistry laboratory supervisors of different hospitals in Shenzhen. The doctors answer only the questions within the scope of their practice and the specialists in laboratory medicine answer all the questions. The proportion of the severity of harm classification was statistically summarized and submitted to the FMEA team for discussion. To draw laboratory’s attention to the risk, the test without consensus received a higher level of risk assessment. Intended use of tests referred to the expert advisor, application guide, or reagent manual and categorized using diagnostic, screening, and patient management decisions.

A modified FMEA was applied to produce an analytic risk rating based on three novel factors, each test of which was graded as follows: 1) Sigma metrics; 2) the severity of harm; 3) intended use (diagnosis, screening, patient management decision). Three novel factors were in accordance with the 5 point system, as shown in Table 2. By multiplying the score of Sigma metrics by the score of severity of harm by the score of intended use, each was assigned a typical RPN. When a test had a different intended use in different clinical applications, it was classified according to the use with the highest risk score. RPN > 50 was considered high risk, the degree of risk was unacceptable; 25 < RPN ≤ 50 for the medium risk, laboratory personnel needed to pay attention to the test; and RPN ≤ 25 for low risk was here considered acceptable. According to the intended use and the accumulated score of the severity of harm, the sigma performance expectations were calculated.

**Table 2:** The risk score of three novel factors

**Risk score**	**Sigma metrics**	**Severity**	**Intended uses**
5	σ < 3	catastrophic	diagnosis
4	3 ≤ σ < 4	critical	screening
3	4 ≤ σ < 5	serious	management
2	5 ≤ σ < 6	minor	-
1	σ ≥ 6	negligible	-

## Results

### Sigma metrics at the critical decision levels

The 42 clinical chemical analytes were performed on five instruments. The results of the Passing-Bablok regression and 95% confidence intervals (CI) for slope and intercept are listed in Table 1. The tests whose 95% CI for slope do not include 1 were as follows: ALT, AST, ALP, BT, Alb, AMY, TG, HDL, Ca, Mg, C3, CRP, cTn-T, pH, pO2. Most tests also had the 95% confidence interval of the y-axis intercept including zero; except for ALB, BT, HDL-C, Cl, Ca, Mg, Fe, PA, pO2, HbA1c.

**Figure 1:**
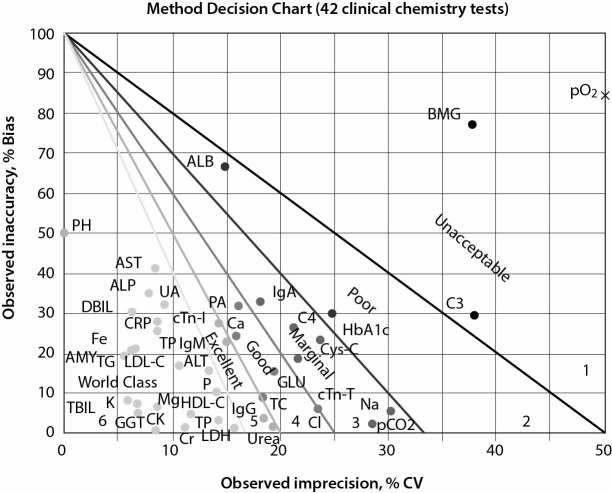
Normalized method decision chart demonstrating the Sigma metrics. ALT - alanine aminotransferase. AST - aspartate aminotransferase. ALP - alkaline phosphatase. GGT - gamma-glutamyltransferase. TP - total protein. ALB – albumin. BT – bilirubin, total. BD – bilirubin, direct. CREA – creatinine. UA - uric acid. Glc – glucose. CK - creatinine kinase. LD - lactate dehydrogenase. AMY – amylase. TG – triglycerides. CHOL - cholesterol. HDL – high density lipoprotein. LDL – low density lipoprotein. K – potassium. Na – sodium. Cl – chloride. Ca – calcium. Mg – magnesium. Phos – inorganic phosphate. Fe – iron. TRSF - transferrin. IgG – immunoglobulin G. IgM – immunoglobulin M. IgA – immunoglobulin A. PA - pre-albumin. Cys-C - cystatin C. BMG - ß2-microglobulin. C3 – complement C3. C4 – complement C4. CRP - C-reactive protein. cTn-I – cardiac troponin I. cTn-T – cardiac troponin T. pH – acidity. pCO2 - partial pressure of carbon dioxide. pO2 - partial pressure of oxygen. HbA1c – glycated haemoglobin.

The Sigma metrics for the critical decision level-making was calculated and listed in Table 1 and the normalized method decision chart demonstrating the sigma values was showed in Figure 1. There were 21 analytes with world class performance (*σ* ≥ 6). The analytes with excellent performance (5 ≤ *σ* < 6) were urea, IgG, IgM, cTn-I, pH; the analytes with good performance (4 ≤ *σ* < 5) were Glc, CHOL, Cl, Ca, PA; the analytes with marginal performance were Na, IgA, C_4_, Cys-C, cTn-T, PCO_2_ and the analytes with poor or unacceptable performance (*σ* ≤ 3) were Alb, C3, BMG, pO2, HbA1c.

### Risk analysis and assessment

A total of 52 professional personal participated in the questionnaire survey, which included 32 doctors, 12 laboratory technicians and 8 clinical biochemistry laboratory supervisors. The number of negligible, minor, serious, critical, and catastrophic was 3, 13, 14, 9 and 3, respectively; the number of diagnostic, screening, and patient management decisions tests was 14, 11, and 17, respectively. There were 7 tests including Glc, Na, Ca, BMG, cTn-T, PCO_2_ and PO_2_ with high-risk of RPN > 50; 8 medium risk items with 25 < RPN ≤ 50. The 5 tests with *σ* < 3 were evaluated as high risk or medium risk items. All of these results were shown in Table 1.

### Establishing a differential sigma performance expectations

Here, 13 tests had sigma performance expectations ≥ 6; 15 tests had sigma performance expectations ≥ 5; 3 tests had sigma performance expectations ≥ 4; 11 tests had sigma performance expectations ≥ 3. The results were shown in detail in Table 3.

**Table 3:** Sigma performance expectations based on severity and intended use classification

**Severity**	**Intended use**	**Test**	**Sigma performance expectations**
**Catastrophic**	Diagnosis	K, cTn-T, cTn-I	σ ≥ 6
	Screening	-	σ ≥ 6
	Management	-	σ ≥ 6
**Critical**	Diagnosis	CREA, Glc, AMY, Na, Ca, pH, PCO_2_, PO_2_	*σ* ≥ 6
	Screening	BT	*σ* ≥ 6
	Management	-	*σ* ≥ 5
**Serious**	Diagnosis	UA	*σ* ≥ 6
	Screening	ALT, AST, BD, Urea, CK, BMG, Cys-C	*σ* ≥ 5
	Management	Cl, Mg, Phos, C3, C4, HbA1c	*σ* ≥ 5
**Minor**	Diagnosis	Fe, TRSF	*σ* ≥ 5
	Screening	GGT, ALP, LD	*σ* ≥ 4
	Management	TP, Alb, TG, CHOL, LDL, IgG, IgM, IgA	*σ* ≥ 3
**Negligible**	Diagnosis	-	*σ* ≥ 3
	Screening	-	*σ* ≥ 3
	Management	HDL, PA, CRP	*σ* ≥ 3
ALT - alanine aminotransferase. AST - aspartate aminotransferase. ALP - alkaline phosphatase. GGT - gamma-glutamyltransferase. TP - total protein. ALB – albumin. BT – bilirubin, total. BD – bilirubin, direct. CREA – creatinine. UA - uric acid. Glc – glucose. CK - creatinine kinase. LD - lactate dehydrogenase. AMY – amylase. TG – triglycerides. CHOL - cholesterol. HDL – high density lipoprotein cholesterol. LDL – low density lipoprotein cholesterol. K – potassium. Na – sodium. Cl – chloride. Ca – calcium. Mg – magnesium. Phos – inorganic phosphate. Fe – iron. TRSF - transferrin. IgG – immunoglobulin G. IgM – immunoglobulin M. IgA – immunoglobulin A. PA - pre-albumin. Cys-C - cystatin C. BMG - ß2-microglobulin. C3 – complement C3. C4 – complement C4. CRP - C-reactive protein. cTn-I – cardiac troponin I. cTn-T – cardiac troponin T. pH – acidity. pCO2 - partial pressure of carbon dioxide. pO_2_ - partial pressure of oxygen. HbA1c – glycated haemoglobin.			

## Discussion

When assessing quality on the *σ* scale, the higher the *σ* metric, the better the quality. Here, quality was assessed on the *σ* scale with a benchmark for minimum process performance of 3*σ* and a goal for world-class quality of 6*σ* (*14*). There were 21 tests with *σ* ≥ 6 and 5 tests with *σ* ≤ 3 of the 42 tests in this study. When calculating Sigma metrics, the selection of appropriate TEa and analyte concentration is crucial. A study in Belgium showed the Sigma metrics of Alb ranged from 1.3 to 32 varied with analyte concentration and the TEa target (*15*). It is desirable that TEa is defined by the highest possible hierarchical model, and then, simple point estimates of sigma at medical decision concentrations are sufficient for laboratory applications (*16*, *17*). However, outcome-based approaches for goal setting may not be possible to set for all analytes (*18*). In this study, the TEa specifications were obtained from the SVP and the EQA criteria from NCCL of China; the CV values and bias were estimated at the critical decision level and the Sigma metrics at that level was calculated.

The integration of RPN of this study is based on three novel factors of Sigma metrics, the severity and intended use. Sigma metrics are directly related to the probability of risk and they can also be indirectly associated with the detection capability of 6 sigma QC rules. Thus, the use of Sigma metrics directly determined the probability of occurrence, simplifying the process of risk assessment. The evaluation of the severity is usually highly subjective and ultimately depends on the team’s experience and competence. So, the summarized data of the survey collected from clinicians and technicians in this study is benefit to making a relatively objective evaluation. Accounting for the intended use of test will also help design a comprehensive risk assessment model. For example, when HbA1c *σ* = 2.8, HbA1c is mainly used as patient management decisions in China, so RPN score of 45 is moderate risk. However, HbA1c was approved by the American Diabetes Association for use as a diagnostic indicator of diabetes, and the RPN score would therefore be adjusted to 75, which is high risk.

In this study, bias was estimated by the EQA data. However, it is several limited, such as the acceptance criteria and peer group comparison, compared to the primary method using a reference standard material (*19*). The intended use of the test is mainly based on the expert advisor, application guide, or reagent manual. Some of them may lack clear criteria. These problems and their solutions still need to be explored and further standardized.

At present, clinical laboratories can’t achieve world class quality (*σ* ≥ 6) for all tests. The results of the risk assessment also showed that tests that posed negligible risk to the patient could be allowed to reach lower Sigma metrics. Identifying the differentiated sigma performance expectations can avoid repeated residual risk evaluation, which is regarded as a time-consuming task (*8*, *9*). If one test can’t achieve the sigma quality performance, it should be adjusted or changed. If intended use lowers the PRN so that the “Sigma performance expectation” isn’t 6 but is only 3 or 4, that still needs to be aligned with the QC procedures implemented. Currently, a test with 3 sigma or below will need more sensitive QC rules, testing multiple QC samples at each QC event, and more frequent QC events to reducing patient risk (*5*, *20*-*22*).

In conclusion, this study demonstrates that the implications of Sigma metrics can be extended beyond the QC design and method acceptability. A new RPN based on Sigma metrics and intended use have been explored, which can make a more comprehensive and objective assessment of the risk of tests. Such model can also be used to establish the Sigma performance expectations and meet the low risk requirements of patients and clinicians.
